# Feasibility Testing a Meditation App for Professionals Working With Youth in the Legal System: Protocol for a Hybrid Type 2 Effectiveness-Implementation Pilot Randomized Controlled Trial

**DOI:** 10.2196/71867

**Published:** 2025-04-24

**Authors:** Ashley D Kendall, Emily Pela, Danielle Amonica, Erin Jaworski, Brenikki Floyd, The AIM+ Community Advisory Board

**Affiliations:** 1 Center for Dissemination and Implementation Science Department of Medicine University of Illinois Chicago Chicago, IL United States; 2 Institute for Health Research and Policy University of Illinois Chicago Chicago, IL United States; 3 Office of the Vice Chancellor for Research University of Illinois Chicago Chicago, IL United States; 4 School of Public Health Community Health Sciences University of Illinois Chicago Chicago, IL United States; 5 See Acknowledgements

**Keywords:** mobile app, mHealth, digital mental health, meditation, mindfulness, juvenile legal system, officers, workplace, ecological momentary assessment (EMA), emotion regulation

## Abstract

**Background:**

Probation officers and other professionals who work with youth in the legal system often experience high chronic workplace stress, which can contribute over time to elevations in anxiety, depression, and workplace burnout. Emotion dysregulation appears to function as a common mechanism underlying these elevations, and growing evidence suggests it can be improved with mindfulness meditation. Implemented successfully, app-based meditation programs could provide professionals with real-time tools for mitigating the effects of chronic workplace stress.

**Objective:**

This paper describes the protocol for a hybrid type 2 effectiveness-implementation pilot randomized controlled trial (RCT) of Bodhi AIM+, a meditation app adapted with and for professionals who work with youth in the legal system. The adaptation process and implementation plan, as well as the pilot RCT design, were guided by theoretically driven implementation science frameworks. The primary outcome of the pilot RCT is app adherence (ie, ongoing app usage per objective analytics data).

**Methods:**

The RCT will be fully remote. Officers and other professionals who work with youth in the legal system (*N*=50) will be individually randomized to use the meditation app or an active control app matched for time and structure. All participants will be asked to follow a 30-day path of brief audio- or video-guided content and invited to use additional app features as desired. In-app analytics will capture the objective usage of each feature. An adaptive engagement design will be employed to engage nonusers of both apps, whereby analytics data indicating nonuse will trigger additional support (eg, text messages promoting engagement). Mental health outcomes and potential moderators and covariates will be self-reported at baseline, posttest, and 6 months. Participants will also complete 1-week bursts of ecological momentary assessment (EMA) at baseline and over the last week of the intervention to capture the mechanistic target (ie, emotion regulation) in real time. All participants will be invited to complete qualitative posttest interviews. Descriptive statistics will be calculated for quantitative data. Qualitative data will be analyzed using a combined deductive-inductive approach. The quantitative and qualitative data will be incorporated into a mixed methods triangulation design, allowing for the evaluation of app adherence and other implementation outcomes as well as related barriers and facilitators to implementation.

**Results:**

Enrollment into the trial started in December 2024 and is currently underway. Study results are anticipated to be available in 2026.

**Conclusions:**

Completion of this pilot trial will inform a future, fully powered RCT to formally evaluate the effectiveness and implementation of Bodhi AIM+. Its use of implementation science methods, coupled with digital technology, positions the present study not only to help make meditation tools available to an important workforce at scale but also to inform broader efforts at implementing and evaluating health apps within workplace settings.

**Trial Registration:**

ClincialTrials.gov NCT06555172; https://clinicaltrials.gov/study/NCT06555172

**International Registered Report Identifier (IRRID):**

DERR1-10.2196/71867

## Introduction

Juvenile probation officers and other professionals who work with youth in the legal system often experience high levels of chronic workplace stress [1,2]. These stressors can include managing large caseloads, supporting youth through traumatic events, and navigating tense home and court visits [1,3]. Many professionals say these stressors can wear on their mental health, contributing over time to elevations in anxiety, depression, and workplace burnout [2,4,5].

Emotion dysregulation (eg, difficulty up-regulating positive emotions or down-regulating negative emotions) appears to be a common mechanism underlying depression, anxiety, and burnout, and it can be improved through mindfulness meditation [6-9]. Moreover, many professionals who work with young people in the legal system express interest in meditation practice [10]. Yet research in this area is sparse, and the scalability of existing efforts has been limited. For example, an early pilot trial indicated that in-person, group-based meditation was acceptable and generally feasible among officers in the juvenile legal system [10]. However, in-person groups are resource-intensive, face limited instructor availability, and often conflict with the shifting demands of officers’ daily schedules. In light of growing evidence that meditation can be effectively delivered via smartphone app [11-13], app-based programs may offer a promising tool for overcoming these barriers. To date, however, no studies of which we are aware have systematically evaluated the effectiveness and implementation of an app-based meditation program with officers and other professionals who support youth in the legal system. This study is a carefully designed first step toward addressing this gap.

It is worth highlighting that individual-level interventions such as meditation do not address the structural and environmental factors that contribute to chronic workplace stress [5,14]. There is thus ultimately a need for programs that intervene directly on these factors. Nonetheless, individual-level interventions can play important roles in providing professionals with tools for more effectively navigating workplace stressors and helping offset potential negative effects such as anxiety, depression, and burnout.

The present study was born out of an earlier project adapting, implementing, and feasibility testing a mindfulness meditation app for youth on probation funded by the National Institute on Drug Abuse (NIDA; grant K99/R00DA047890). The setting for both projects is Cook County, which encompasses Chicago and its surrounding suburbs and houses one of the largest legal systems in the United States. Throughout our pilot trial of the meditation app for youth, officers approached our team requesting their own version of the app both (1) as a stress reduction tool for themselves during the workday, and (2) as a resource to support mindfulness skills–building among youth on their caseloads. In response to these requests, we developed a grant proposal for two main phases of research activity. The grant was funded by the National Center for Complementary and Integrative Health (NCCIH; grant R34AT012078).

During the first phase of the activity, which was conducted in preparation for the pilot trial detailed in this protocol paper (ie, the second phase), we partnered with officers in the juvenile legal system and other stakeholders to guide the adaptation and implementation of an existing meditation app, Bodhi, for use by officers in the workplace. During this process, we received feedback that other professionals working with youth in the legal system, for example in independently contracted probation programming, faced similar workplace stressors and had a shared interest in a workplace meditation app. We thus adapted Bodhi with the intention that it could be used not only by officers but also by other related professionals more broadly.

The original Bodhi app was developed by Tibetan Buddhist meditation teachers to deliver authentic meditation practices in a modern and intuitive interface based on extensive user testing. The meditation practices were derived from the Nyingma lineage, the oldest of the four major schools of Tibetan Buddhism. The original app featured a 30-day program to help users build a daily meditation practice through brief audio-guided meditations, complemented by short videos from scientific experts and everyday meditators. Bodhi also included a menu of To Go integration meditations that users could select to prepare for specific situations (eg, flying on an airplane). Youth from our previous study named their adapted app Bodhi AIM (Action In Mindfulness); officers voted to name the companion version adapted for their use Bodhi AIM+ (called AIM+ for short).

The process of adapting and implementing Bodhi for officers and other professionals was guided by the EPIS (Exploration Preparation Implementation Sustainment) and CFIR (Consolidated Framework for Implementation Research) implementation science frameworks [15-17]. We collaborated with stakeholders to identify key determinants (ie, barriers and facilitators) of professionals’ adherence to the app and develop corresponding implementation strategies to promote adherence. Adherence (ie, ongoing app usage) was identified as the primary outcome because low adherence to health apps is a pervasive issue and adequate levels are a precondition to achieving health benefits [18]. The implementation strategies that were developed included tailoring the meditation practices for relevance to officers and other professionals during their workday (eg, practices for before a meeting or before court), recording the practices with a guide stakeholders found appealing and trustworthy, and building an automated adaptive engagement design to detect low app adherence based on analytics data and trigger additional support (eg, text messages, as detailed below). All of this was accomplished through an iterative series of qualitative interviews with officers rooted in the EPIS and CFIR frameworks. These formal interviews were supplemented by ongoing feedback from a range of other stakeholders including leadership in the juvenile legal system, youth with experience in the legal system, meditation experts, and the AIM+ Community Advisory Board (CAB). The AIM+ CAB comprised adults with backgrounds relevant to studying AIM+ including working in the juvenile legal system, youth services, meditation instruction, and community mental health.

For the second phase of activity, we will conduct a hybrid type 2 pilot randomized controlled trial (RCT) of AIM+ with officers and other professionals working with youth in the legal system. Hybrid type 2 designs, which address both effectiveness and implementation outcomes, are appropriate when the determinants of implementing an intervention have already been identified and data on corresponding implementation strategies are available [19,20]. In the case of AIM+, we used the first phase of our project activities, described above, to meet these requirements. We selected effectiveness over efficacy testing consistent with the increasing consensus that when the safety concerns for intervention are low, the intervention should be tested in the real-world contexts in which it is meant to be delivered [21]. Compared with efficacy testing, this approach enhances insight into real-world implementation and expedites realization of the intervention’s public health impact [21]. The present paper details the protocol for this hybrid type 2 pilot RCT design.

## Methods

### Overview

**Figure 1:**
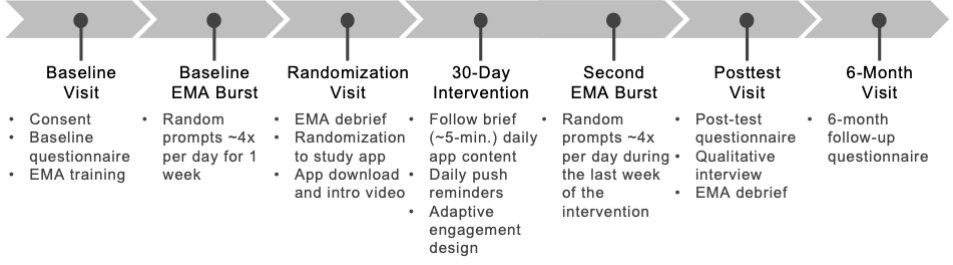
Overview of the hybrid type 2 pilot randomized controlled trial procedures. EMA: ecological momentary assessment; min.: minute.

We aim to enroll 50 professionals working with youth in the legal system into the pilot RCT. The sample size was selected pragmatically to identify issues related to the feasibility of implementing AIM across both quantitative indicators (eg, objective app adherence) and qualitative feedback (ie, achieving thematic saturation in the interview data). Once enrolled, participants’ RCT activities will last approximately 6 months; see Figure 1 for an overview. All study visits will take place remotely via phone or a secure videoconferencing platform. Study data will be collected and managed using REDCap (Research Electronic Data Capture; Vanderbilt University) tools hosted at the University of Illinois Chicago (UIC). REDCap is a secure, web-based software platform designed to support data capture, tracking, storage, integration, and interoperability with external sources [22,23].

### Ethical Considerations

#### Ethics Approval

The study protocol was approved by an independent monitoring committee (IMC) of scientific experts constituted by the study team to advise the sponsor and study investigators, the AIM+ CAB, and the UIC institutional review board (IRB; protocol # 2023-0363). Details regarding the IMC can be found at nccih.nih.gov. The trial is registered on ClincialTrials.gov (NCT06555172).

#### Centralized Management of Study Procedures and Data

The data manager developed a robust REDCap infrastructure that will support and automate study procedures including recruitment, enrollment, randomization, retention, assessment, and data management. Informed consent will be obtained electronically by study staff via REDCap; participants’ ability to opt out of the study at any point will be emphasized. The REDCap system will also enable secure, remote administration of the study assessments. Processes were programmed into REDCap to validate and check for missing data in real time. Regarding app analytics data, date and time stamps indicating when participants open and close each feature in their study app will be collected and initially stored on a secure server. Other than the app analytics data, the study apps will not access, record, or store any information from participants’ phones. Research staff will download coded app analytics data off the server without identifiers; these coded data will be stored on secure UIC servers and imported as relevant into REDCap. Scripts will clean, score, and store coded data separate from identifiers. REDCap will track all participant contact and autogenerate a CONSORT (Consolidated Standards of Reporting Trials) map and progress reports including enrollment tables, retention rates, adverse events, quality assurances, and project workflow. This will help ensure that inclusion and exclusion criteria are met, all compliance regulations are met, and the data are collected accurately and completely. Any unanticipated problems, including adverse events, will be documented in REDCap and reported to the IRB, IMC, and sponsor. Trial conduct will be audited at least monthly by the principal investigator, and at least annually by the IRB, IMC, and sponsor. Important protocol modifications are not anticipated but will be reviewed by the IRB, IMC, and sponsor before implementation and will be updated within ClinicalTrials.gov.

#### Compensation

Participants will be compensated via electronic payment consistent with standards in the field: US $45, US $50, and US $55 for the baseline, posttest, and 6-month follow-up questionnaires, respectively; US $10 for app download and orientation; US $50 for participation in each EMA burst and debrief, with a US $25 bonus at each burst for completion of at least 80% of the EMA reports; and US $30 for the posttest qualitative interview. We anticipate that employment policy will prohibit compensation for some participants; we will carefully document compensation eligibility to enable preliminary examination of its associations with study outcomes. In addition to the monetary incentives for eligible participants, all participants will be offered the option to receive a personalized summary based on their data at the end of the study to encourage ongoing participation.

### Participants

Participants will be recruited from Cook County Juvenile Probation and Court Services and related programming for young people in the legal system. All participants must be (1) currently working with youth who are involved in the legal system, (2) English-speaking due to currently available study instruments, (3) able to understand and provide consent, and (4) have an Android or Apple smartphone.

### App-Based Interventions

#### Bodhi AIM+

**Figure 2:**
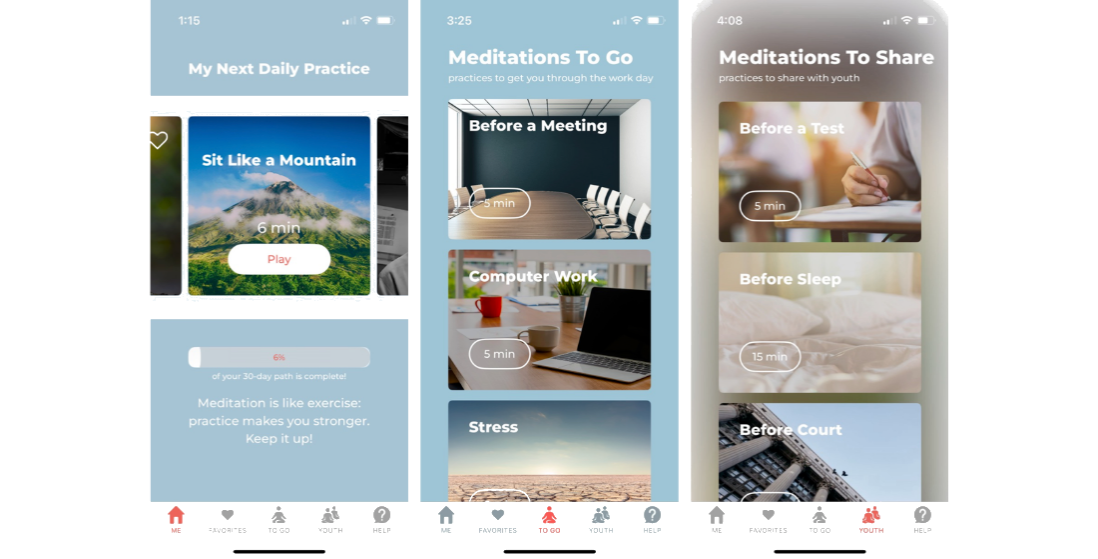
Screenshots of the Bodhi AIM+ prototype, including the 30-day meditation path (left), meditations to go (center), and meditations to share with youth (right). AIM+: Action In Mindfulness.

The AIM+ app—officially named Bodhi AIM+ to recognize its connection to the Bodhi app from which it was adapted—is a meditation app for professionals working with youth in the legal system. It includes 5 main parts: Home, Favorites, To Go, Youth, and Help (see Figure 2 for prototype screenshots). The Home page features a 30-day path of brief (approximately 5- to 10-minute) audio-guided meditations with videos interspersed to promote engagement. Users are encouraged to follow 1 of the practices each day for a month. To unlock the next practice, a user must first play the current practice to completion; once unlocked, users can scroll forward or backward to replay practices as desired. They can also tap a heart icon on any of the daily path practices; these practices will then appear in the Favorites part of their app for easy access. In addition to following the 30-day path, users can select from a menu of To Go audio-guided meditations to prepare for specific situations (eg, attending a meeting). Users also have access to a menu of To Go audio-guided meditations designed with and for youth in the legal system and featured in the original AIM app for young people (eg, meditations for before going to a party or to court). Users are invited to play these practices for any youth who might find them beneficial. Finally, the Help tab in AIM+ serves 2 main functions. First, users can use this part of the app to contact study staff by phone or text for support. Second, the Help tab includes referral buttons for users to share opportunities to participate in clinical trials research of AIM+ and AIM with colleagues and youth, respectively. These buttons auto-populate text messages that provide the recipient with a link to the relevant IRB-approved study flier where they are presented with more information. Users are instructed not to inquire further with peers or youth if they decide to participate to minimize the potential for coercion.

#### Resource+

The Resource+ app is matched to AIM+ for structure and time. The user interfaces are identical—both made up of Home, Favorites, To Go, Youth, and Help pages—with audio and video files occurring in the same sequence and each lasting approximately the same time across apps. However, whereas AIM+ features meditation practices to improve emotion regulation, Resource+ presents educational overviews of resources for youth to enhance professionals’ knowledge of available resources for their clients. Each day of the 30-day path showcases a new resource; popular resources are also presented on the To Go page for easy reference. The Youth page includes overviews of resources intended to be played directly to any young people for whom they might be relevant. The Help page is identical to that in AIM+.

### Hybrid Type 2 Pilot RCT

#### Recruitment

To recruit professionals who work with youth in the legal system into the pilot RCT, 3 main methods may be used: (1) live presentations by study staff, (2) email or flyer distribution, and (3) in-app peer referral. Presentations may be held in-person or virtually and will include an overview of the study along with time for questions. Emails and flyers may be distributed via relevant workplace contacts and will include a brief overview of the study, including a link to a video for learning more. In-app peer referrals will generate a prepopulated text message with a link to the same video for learning about the study. This text message will emphasize that the person sending it will not know if the person who receives it chooses to participate or not. Throughout the recruitment procedures, it will be explicitly stated that participation is voluntary, can be terminated at any time, and will not impact participants’ standing within their workplace, UIC, or any related institutions. Interested participants will be invited to submit their contact information via a secure REDCap link. Study staff will follow up to complete an eligibility screener and conduct the baseline visit.

#### Baseline Visit

Potential participants will be asked to meet remotely via phone or a secure video platform at a time that is convenient for them in a private location. Once consent has been obtained, study staff will send a secure link to complete the baseline questionnaire on the participant’s phone or computer. It will probe health outcomes along with potential moderators and covariates, as described below. Staff will remain on the call to answer participants’ questions (eg, about the meaning of words) but will not advise on how participants answer or see participants’ answers.

After the baseline questionnaire, the study staff member will provide training in ecological momentary assessment (EMA). EMA involves having people report on their experiences in real time throughout their daily lives [24,25], making it ideally suited to capturing emotion regulation (ie, the mechanistic target of the AIM+ app). All participants will be asked to complete EMA reports on their cell phones in response to random prompts approximately 4 times per day over 1 week from 9 AM to 9 PM. Each report will probe current affective states and attentional control as well as objective contexts (ie, if it is during or outside the workday along with current location, activity, and companionship). Participants will be instructed to respond to each prompt as quickly as possible without interfering with their job (eg, while in a meeting), creating safety concerns (eg, while driving), or causing potential embarrassment (eg, while in religious services). Each participant will complete a mock EMA report and go over any questions they have. They will be asked to predict their schedule for the upcoming week; based on this information, staff will guide each participant in anticipating obstacles to reporting and potential solutions.

#### Baseline EMA Burst

The baseline EMA burst is intended to begin with completion of the baseline visit and continue for the next week (ie, for a total of 7 consecutive days). Each day, participants will be prompted to complete approximately 4 EMA reports at random intervals between 9 AM and 9 PM. On EMA days 2 and 4, participants will be sent text messages updating them on their progress (eg, percentage of EMA reports completed out of the prompts sent) and inviting them to contact staff with any questions. Staff will remain available over the week during business hours. All EMA reports will be automatically date- and time-stamped and stored on a secure cloud server rather than on participants’ local devices.

#### Randomization Visit

Study staff will meet again virtually with each participant after the baseline EMA burst. Staff will review the total percentage of EMA surveys completed and discuss any issues with compliance, including any potential solutions for the second EMA burst based on their experience. Participants will then be individually randomized via REDCap to the AIM+ or Resource+ app at a 1:1 ratio. The allocation sequence will be generated in advance by the data manager under the supervision of a biostatistician and loaded into REDCap, where it will be fully concealed from the research team. Research staff will use the randomization module in REDCap to assign allocations, but will not be able to see the schedule or view allocations until they are assigned. Both staff and participants will thus be blind to arm through the completion of all baseline assessments. Next, staff will guide participants in downloading their relevant app and playing Day 1 of the 30-day path, which provides a video-guided introduction to the app. Participants will be encouraged to follow the 30-day path by playing a brief path file each day, and invited to use the other app features as desired; for details on the app content, see the AIM+ and Resource+ descriptions above. Participants will be trained not to use their app when doing so could interfere with work, be dangerous, or be embarrassing. Staff will guide participants in planning for when they will be most successful in using the app and then schedule personalized daily in-app push reminders based on those times.

#### Intervention With an Adaptive Engagement Design

Participants will be asked to use their app daily for 30 days. Adherence to the intervention (ie, ongoing app usage) will be promoted via 2 main methods. First, all participants will receive daily in-app push reminders to use the app as described above. Second, adherence will be automatically monitored, and additional support will be triggered via an adaptive engagement design: after any 2 consecutive days of nonuse, participants will be sent a text message encouraging re-engagement with the app. After approximately 1 week of nonuse, participants will receive a phone call from staff on the closest business day, during which staff will help identify and troubleshoot barriers to adherence. This pattern will repeat for up to approximately 2 weeks, at which point there will be a final phone call attempt before discontinuing texts and calls to reduce the potential for unwanted burden.

#### Second EMA Burst

In advance of the final intervention week, study staff will contact each participant to provide instructions for completing their second EMA burst, which will be scheduled to coincide as closely as possible with the fourth week of the intervention. Staff will meet virtually with participants to refresh their EMA training, including reviewing any barriers and facilitators to EMA adherence identified after their baseline burst. The second EMA burst is intended to begin immediately after the call and continue for 7 days. It will follow the same procedures as at baseline.

#### Posttest Visit

Participants will meet virtually with study staff for a posttest visit occurring approximately 1 month after the start of app use (ie, as close as possible to the end of the 30-day app path). This will mirror the baseline visit procedures except that (1) the posttest questionnaire will measure health outcomes and potential covariates since the last assessment and will include an assessment of implementation outcomes, (2) there will be a posttest qualitative interview, and (3) there will be a debrief of the second EMA burst, during which staff solicit feedback on any refinements participants suggest to the procedures.

The posttest qualitative interview will probe 3 main domains: app implementation (eg, determinants of app adherence, acceptability, and penetration), any perceived effects of the app, and suggestions for future refinements. Wherever possible, questions will be worded identically across arms. Staff will be trained to conduct the interviews through didactic instruction, role-playing, and supervised practice, focusing on techniques such as open-ended questions, active listening, and probing for depth while minimizing interviewer bias [26]. Proficiency will be assessed through mock interviews and iterative feedback. The interviews will be audio recorded, securely stored, and transcribed for qualitative coding. At the conclusion of the interview, staff will invite the participant to retain the app on their phone until the 6-month follow-up. All participants will receive automated text messages from the study team once monthly to promote retention.

#### 6-Month Follow-Up Visit

The remote 6-month follow-up visit will be comprised only of the 6-month follow-up questionnaire. The questionnaire will be administered via REDCap using the same procedures as during the baseline and posttest visits. It will measure health outcomes and potential covariates since the last assessment.

### Outcomes

Both quantitative and qualitative data will be collected. Table 1 summarizes the primary and secondary quantitative outcomes along with potential moderators and covariates. As described above, qualitative themes that will be probed in posttest interviews to complement and contextualize the quantitative data will include determinants of app implementation, perceived effects of the apps, and suggestions for future refinements.

**Table 1:** Quantitative outcomes for the hybrid type 2 pilot randomized controlled trial (RCT).

Outcome				Measure		Time point		Source
**Primary outcome**								
	App adherence		Days of objective app usage		Intervention		App analytics	
**Secondary implementation outcomes**								
		Acceptability	Acceptability of Intervention Measure (AIM) [27]		1 month		Questionnaire	
		Appropriateness	Intervention Appropriateness Measure (IAM) [27]		1 month		Questionnaire	
		Feasibility	Feasibility of Intervention Measure (FIM) [27]		1 month		Questionnaire	
		Usability	System Usability Scale (SUS) [28]		1 month		Questionnaire	
		Satisfaction	Client Satisfaction Questionnaire CSQ-8) [29]		1 month		Questionnaire	
		Penetration	In-app referral to AIM+ RCT^a^		Intervention		App analytics	
		Penetration-youth	In-app referral to AIM RCT for youth		Intervention		App analytics	
		Penetration-youth	Files for youth played in AIM+ or Resource+		Intervention		App analytics	
**Secondary health outcomes**								
		Anxiety	PROMIS^b^ v1.0 Anxiety (standard computer adaptive test) [30]		Baseline, 1 month, 6 months^c^		Questionnaire	
		Depression	PROMIS v1.0 Depression (standard computer adaptive test) [30]		Baseline, 1 month, 6 months		Questionnaire	
		Workplace burnout	Maslach Burnout Inventory–General Survey (MBI-GS) [31]		Baseline, 1 month, 6 months		Questionnaire	
		Perceived stress	Perceived Stress Scale (PSS) [32]		Baseline, 1 month, 6 months		Questionnaire	
		Anger	PROMIS Short Form v1.1 Anger [30]		Baseline, 1 month, 6 months		Questionnaire	
		Sleep disturbance	PROMIS Short Form v1.0 Sleep Disturbance [33]		Baseline, 1 month, 6 months		Questionnaire	
		Alcohol use	PROMIS v1.0 Alcohol Use [34,35]		Baseline, 1 month, 6 months		Questionnaire	
		Resilience	Brief Resilience Scale (BRS) [36]		Baseline, 1 month, 6 months		Questionnaire	
		Mindfulness	Five Facets of Mindfulness Questionnaire (FFMQ-15) [37-39]		Baseline, 1 month, 6 months		Questionnaire	
		Attentional control	Attentional Control Scale (ACS) [40]		Baseline, 1 month, 6 months		Questionnaire	
		Emotion regulation	Negative Mood Regulation (NMR) [41]		Baseline, 1 month, 6 months		Questionnaire	
		Current mood states	International Positive Affect Negative Affect Schedule Short Form (I-PANAS-SF) [42,43]		Baseline, last intervention week		EMA^d^	
		Current attention	2 items adapted from Chin et al [44]		Baseline, last intervention week		EMA	
**Moderators and covariates**								
	Demographics		Gender, age, race and ethnicity, education, household income		Baseline		Questionnaire	
	Employment		Length of current employment, role		Baseline		Questionnaire	
	Mental health treatment history		Psychotherapy, hospitalization, psychotropic medication		Baseline, 1 month, 6 months		Questionnaire	
	Other meditation		Meditation practice frequency and type		Baseline, 1 month, 6 months		Questionnaire	

^a^RCT: randomized controlled trial.

^b^PROMIS: Patient-Reported Outcomes Measurement Information System.

^c^1 month and 6 months: 1-month follow-up (ie, posttest) and 6-month follow-up, respectively.

^d^EMA: ecological momentary assessment.

The primary outcome of the pilot RCT will be app adherence per objective analytics data. A total of 2 main operationalizations will be considered. First, following other pilot RCTs of health apps [45], we aim for >60% of participants to open the app on at least 1 day after their randomization visit. Second, we will examine the mean number of content files played over the course of the intervention. Although there is no established dosage for app-based meditation [46], meditation studies have observed positive effects on mental health after 2 weeks, including for brief, audio-guided mindfulness practices [47]. Accordingly, we will explore if participants complete an average of 14 daily path files, and if this benchmark appears to be met within some subsets of participants but not others to help contextualize the findings and inform future work.

Secondary implementation outcomes will include acceptability, appropriateness, and feasibility as measured by the Acceptability of Intervention Measure (AIM), Intervention Appropriateness Measure (IAM), and Feasibility of Intervention Measure (FIM) [27], respectively; usability as measured by the System Usability Scale (SUS) [28], satisfaction as measured by the Client Satisfaction Questionnaire (CSQ) [29], and penetration as indicated by objective analytics (ie, in-app referral to our RCTs of AIM+ for professionals and AIM for youth, and files played for youth by professionals using their study app).

All health effects will be treated as secondary outcomes. In accordance with our sponsor guidelines, the purpose of this data collection will be to ensure its feasibility in preparation for a future, fully-powered trial if justified by the present design [48]. That future trial would focus on group differences in anxiety and depression symptoms as measured by the Patient-Reported Outcomes Measurement Information System (PROMIS) v1.0 Anxiety and Depression scales, respectively [30], workplace burnout as measured by the Maslach Burnout Inventory-General Survey (MBI-GS) [31], and perceived stress as measured by the Perceived Stress Scale (PSS) [32]. It will also explore changes in anger, sleep disturbance, and alcohol use as measured by the PROMIS Short Form v1.1 Anger [30], PROMIS Short Form v1.0 Sleep Disturbance [33], and PROMIS v1.0 Alcohol Use [34,35] scales; in resilience as measured by the Brief Resilience Scale (BRS) [36]; in mindfulness as measured by the Five Facets of Mindfulness Questionnaire (FFMQ-15) [37-39]; and in attentional control as measured by the Attentional Control Scale (ACS) [40]. The primary mechanistic target—emotion regulation—will be assessed both retrospectively via the Negative Mood Regulation (NMR) scale [41] and in real time via EMA reports that include a short form of the International Positive Affect Negative Affect Schedule (I-PANAS-SF) [42,43]. A real-time assessment of attentional control will also be included in the EMA reports using 2 items adapted from Chin and colleagues [44]. Data collection will be considered feasible if at least 70% of participants complete the questionnaire measures at 1 and 6 months, and if participants fill out an average of at least 70% of the EMA reports at each burst. EMA reports will be counted if they are completed in full. We will also examine partial EMA completion rates during the pilot RCT and, as relevant, probe barriers to fully completing the EMA reports during the qualitative exit interviews with participants.

### Data Analysis

Descriptive statistics will be calculated for the quantitative data (eg, mean number of app files played per objective analytics data, mean total and subscale scores for each self-report measure). We will explore potential biases in missing questionnaires and EMA data (eg, as a function of baseline characteristics). This will be accomplished using chi-square tests for categorical variables and *t* tests or nonparametric Wilcoxon rank-sum tests for continuous variables. Given the relatively small sample size in this pilot study, we will flag potential bias in missing data based in part on the descriptive statistics from these tests, rather than relying only on their significance levels, and use this preliminary information to help inform the interpretation of our results.

Qualitative data will be recorded, transcribed, and analyzed using a combined deductive-inductive approach (ie, use of a priori codes in the early stages, allowing for additional codes to develop from the data [49]). Analytic codes based on a priori categories will correspond to the foci of the interviews and themes that emerge in the data. Coders will follow an iterative procedure of refining, merging, or subdividing codes until a consistent set is developed. The quantitative and qualitative data will be incorporated into a mixed-methods triangulation design following an iterative process based on the recommendations of Turner et al [50], allowing for evaluation of app adherence and other implementation outcomes, as well as related determinants of implementation.

## Results

Enrollment into the pilot RCT began in December 2024 and is currently ongoing; study results are anticipated to be available in 2026.

## Discussion

This hybrid type 2 effectiveness-implementation pilot RCT of the AIM+ meditation app will integrate quantitative and qualitative data to evaluate app adherence (ie, ongoing app usage) and explore other implementation outcomes, as well as related barriers and facilitators to implementation. It will include an in-depth assessment of health outcomes, including real-time capture of the mechanistic target (ie, emotion regulation), allowing for evaluation of the feasibility of this data collection and setting the stage for a fully-powered RCT if justified by the present pilot findings.

The AIM+ meditation app was built with and for officers and other professionals who work with youth in the legal system to make authentic mindfulness meditation practices available throughout the workday. It is a direct response to requests from stakeholders for such an intervention, created in collaboration with our community partners by adapting an existing meditation app, Bodhi, for use by the target audience. The adaptation process and implementation plan—as well as the pilot RCT detailed in this protocol paper—were guided by the EPIS and CFIR implementation science frameworks [15-17].

Consistent with pilot RCTs of other health apps [45], we expect that >60% of participants will open the app at least 1 day after their randomization visit per objective analytics data, and we will explore the mean total number of content files played over the course of the intervention. We further expect that our longitudinal assessments of health outcomes will be feasible, as evidenced by at least 70% of participants completing the questionnaire measures at each follow-up time point and at least 70% of the EMA reports being completed within each week-long EMA burst.

Potential limitations to the study warrant consideration, particularly regarding their implications for a fully-powered trial that could build on this design. First, emotion regulation is the primary mechanistic target of AIM+, yet its retrospective assessment is highly subject to recall bias [51]. To address this, we include EMA assessments of mood, which will provide data on emotion regulation in real time and in participants’ daily lives. Next, it is not possible to blind participants to arm after randomization has occurred. To partially address this, our control app is matched to AIM+ for time and structure. At the same time, given that common factors are estimated to account for a substantial portion of the effects observed in mental health studies [52], this matching is likely to attenuate our ability in the future to formally detect between-group differences in treatment effects. Finally, the nature of our sample—specifically, professionals who work with youth in the legal system—will necessarily limit the generalizability of the results. However, this group represents a key public health demographic, and findings from these professionals are likely to still have some relevance to implementing health apps with other professionals during the workday.

In summary, this work represents an important public health direction given the high levels of chronic workplace stress that professionals who work with youth in the legal system often face [1,2], the relevance of mindfulness meditation to helping mitigate the negative downstream effects of this stress [6-9], and the interest such professionals have expressed in workplace meditation [10]. Although early studies in this area generally supported acceptability and feasibility [10], these in-person initiatives faced key barriers to scalability, constraining their potential for impact on a national level. The use in the present study of theoretically-driven implementation science methods coupled with scalable, digital technology positions this study not only to help make meditation tools available to an important national workforce at scale but also to inform broader efforts at implementing health apps within workplace settings.

## Abbreviations

ACS: Attentional Control Scale
AIM: Acceptability of Intervention Measure
AIM+: Action In Mindfulness
BRS: Brief Resilience Scale
CAB: community advisory board
CFIR: Consolidated Framework for Implementation Research
CONSORT: Consolidated Standards of Reporting Trials
CSQ: Client Satisfaction Questionnaire
EMA: ecological momentary assessment
EPIS: Exploration Preparation Implementation Sustainment
FFMQ-15: Five Facets of Mindfulness Questionnaire
FIM: Feasibility of Intervention Measure
IAM: Intervention Appropriateness Measure
IMC: independent monitoring committee
I-PANAS-SF: International Positive and Negative Affect Schedule Short Form
IRB: institutional review board
MBI-GS: Maslach Burnout Inventory-General Survey
NCCIH: National Center for Complementary and Integrative Health
NIDA: National Institute on Drug Abuse
NMR: Negative Mood Regulation
PROMIS: Patient-Reported Outcomes Measurement Information System
PSS: Perceived Stress Scale
RCT: randomized controlled trial
REDCap: Research Electronic Data Capture
SUS: System Usability Scale
UIC: University of Illinois Chicago

## Data Availability

Data sharing is not applicable to this protocol paper as no finalized data sets have yet been generated.
